# Psychological interventions mitigated occupational stress in high-risk workers in Shenzhen, China

**DOI:** 10.3389/fpubh.2025.1636004

**Published:** 2025-07-18

**Authors:** Naixing Zhang, Wei Zhou, Aipin Xiao, Shaofan Weng, Liuzhuo Zhang, Dexiang Zhu, Jinlin Wang, Ling Nian, Dafeng Lin

**Affiliations:** ^1^Occupational Health Department, Shenzhen Prevention and Treatment Center for Occupational Diseases, Shenzhen, China; ^2^Public Health Department, Southern Medical University, Guangzhou, China; ^3^Psychological Crisis Intervention Department, Shenzhen Mental Health Center & Shenzhen Kangning Hospital, Shenzhen, China

**Keywords:** occupational stress, social support, Job Content Questionnaire, group psychological support session, mental health training

## Abstract

**Introduction:**

Occupational stress has emerged as a significant factor impacting the physical and mental wellbeing of workers in China. This study investigated occupational stress among the potential high-risk workers in Shenzhen, China, and evaluated the psychological interventions subsequently implemented.

**Methods:**

A stratified cluster sampling method was employed, randomly selecting one quarter of clusters (clustered by work unit) from each of the four strata (stratified by occupational category) including firefighters, bus drivers, video display terminal (VDT) operators, and port workers, respectively, as the study cohort. Occupational stress was assessed at baseline and after psychological interventions using the “Job Content Questionnaire.” The interventions primarily included regular occupational mental health training and group psychological support sessions. Baseline occupational stress detection rates and high social support rates were analyzed, along with their post-intervention changes, to comprehensively evaluate the intervention effectiveness.

**Results:**

The cohort comprised 3,237 participants, with a median age of 31 years, 92.18% were male, and 96.14% were Han Chinese. The baseline occupational stress detection rate was 52.73%, and the high social support rate was 21.19%. Comparative analysis revealed statistically significant differences (all *P* < 0.05) in occupational stress detection rates across different age groups, ethnicities, education levels, smoking habits, weekly working hours, and working years. Similarly, high social support rates varied significantly (all *P* < 0.05) by age, education level, marital status, exercise habit, occupation category, weekly working hour, and working year. After 2 years of intervention, the occupational stress detection rate decreased significantly to 47.82% (*P* < 0.001), while the high social support rate (19.68%) showed no significant change. Subgroup analysis indicated significant reductions in occupational stress detection rates (all *P* < 0.05) among individuals aged ≥ 27 years, males, Han Chinese, those with high school or vocational school or higher, married individuals, never or occasional drinkers, firefighters or bus drivers, those working 40–48 or >56 h per week, and those with >6 years of work.

**Conclusions:**

Occupational stress is a prominent issue among firefighters, bus drivers, VDT operators, and port workers in Shenzhen, China. Mental health interventions could effectively reduce occupational stress, demonstrating significant value in improving the psychological wellbeing of high-risk populations.

## Introduction

In modern society, the complexity and diversity of work tasks and environments have made occupational stress a critical factor affecting workers' physical and mental health (1, 2). Occupational stress refers to a series of physiological, psychological, and behavioral stress responses resulting from a mismatch between job demands and an individual's capabilities or resources, as well as influences from the work environment and organizational management (3). Prolonged exposure to occupational stress not only reduces work efficiency and quality of life but may also lead to various physical and mental disorders, such as cardiovascular diseases, anxiety, and depression (4, 5), imposing a substantial burden on individuals, families, and society.

As a special economic zone and an international metropolis in China, Shenzhen hosts a vast workforce. With rapid economic development and increasing competitive pressures, occupational stress among workers has become increasingly prominent. While occupational stress among healthcare workers has received widespread attention (1, 5), that of other professional groups is often overlooked. For instance, firefighters face life-threatening emergencies, unpredictable work schedules, and exposure to traumatic scenes, leading to psychological strain and physical exhaustion (6). Bus drivers endure prolonged concentration, traffic congestion, passenger conflicts, and rigid timetables, contributing to chronic fatigue and emotional burnout (7). Video display terminal (VDT) operators (e.g., office staff, designers) experience eye strain, musculoskeletal disorders from prolonged sitting, and high mental workloads due to repetitive screen-based tasks (8). And port workers cope with heavy machinery hazards, shift work disrupting circadian rhythms, and pressure to meet tight deadlines in noisy, high-risk environments (9). Common stressors across these roles include high responsibility, limited control over work conditions, and inadequate recovery time, all of which may elevate risks of anxiety, depression, or cardiovascular diseases. Understanding the occupational stress levels of the above-mentioned high-risk worker groups, implementing interventions, and evaluating their effectiveness are of significant practical importance for developing targeted occupational health promotion strategies and safeguarding workers' wellbeing. Currently, research on occupational stress in these high-risk populations remains limited, and systematic evaluations of intervention outcomes are lacking. Therefore, conducting this study is of urgent necessity.

## Methods

### Study subjects

The study employed a stratified cluster sampling method. We first stratified the population by occupational category, dividing them into four strata (firefighters, bus drivers, VDT operators, and port workers). Then we divided the population in each stratum into clusters by their work units. One quarter of clusters from each stratum were randomly selected to constitute the study cohort. The inclusion criteria comprised: (1) voluntary participation in psychological interventions, and (2) commitment to completing at least two occupational health investigations. Exclusion criteria were: (1) presence of significant mental and/or organic disorders, and (2) recent use of psychotropic medications. The final cohort included 3,237 eligible participants, all of whom provided written informed consent. The study protocol was approved by the Medical Ethics Committee of Shenzhen Prevention and Treatment Center for Occupational Diseases (*Approval No*. LL2020-34).

### Baseline information and occupational stress investigation

During the baseline occupational health survey of the cohort, questionnaires were used to collect general demographic characteristics, daily lifestyle habits, and work-related information. In this study, smoking was defined as consuming at least one cigarette per day for 6 months or more prior to the survey; alcohol consumption was defined as drinking at least once per week for 6 months or more prior to the survey; regular exercise was defined as engaging in at least moderate-intensity physical activity (resulting in accelerated breathing and heart rate) for ≥30 min per session, twice or more per week, for 6 months or more prior to the survey.

The Job Content Questionnaire (JCQ22) is an internationally recognized tool for assessing occupational stress, with demonstrated reliability and validity (10, 11). This study utilized the Chinese version of JCQ22 to evaluate occupational stress levels in the cohort population at baseline and after receiving mental health interventions. This questionnaire includes 22 items covering three dimensions. These dimensions include five items for job demands, nine items for job control (three items for autonomy decisions and six items for job skills) and eight items for social support (four items for colleague support and four items for superior support). The primary outcome measures were the occupational stress detection rate and high social support rate. The occupational stress detection rate was determined based on JCQ22 questionnaire scores according to established criteria, while the high social support rate was classified according to scores in the social support dimension of the questionnaire. Specifically, the questionnaire uses a 4-point Likert scale, ranging from 1 to 4 and covering responses from “disagree entirely” to “agree entirely.” Stress levels are evaluated using the ratio of the average job demands score to the average job control score (D/L × 9/5). A ratio of >1 indicates the presence of occupational stress. The level of social support is assessed by the total score of social support. Scores above 24 indicate high social support.

### Psychological interventions

The intervention was organized by occupational health experts from Shenzhen Prevention and Treatment Center for Occupational Diseases, with the assistance of trade unions from the participants' work units. It primarily consisted of occupational mental health training and group psychological support sessions, which were conducted and facilitated quarterly at the participants' work units by psychologists/psychotherapists from Shenzhen Mental Health Center & Shenzhen Kangning Hospital. Each intervention activity required at least 80% of participants to attend, with a duration of 60–90 min. The occupational mental health training covered mental health knowledge dissemination, occupational stress coping techniques, and emotion management methods. The group psychological support sessions facilitated communication and mutual support among participants through organized group activities, thereby enhancing their psychological adjustment capabilities. The entire intervention period lasted nearly 2 years (from Mar. 2023 to Dec. 2024).

### Quality control

The general information and occupational stress surveys were both administered using on-site QR code scanning through the “Questionnaire Star” online platform (https://szzfy2020.wjx.cn/vm/mNV361m.aspx#), with standardized training provided to all investigators who supervised the on-site data collection process. The investigators verified and corrected questionnaires with missing items, incomplete responses, or irregular entries. Questionnaires containing more than 10% missing items or logical inconsistencies were excluded from the analysis.

### Statistical analysis

The collected data were statistically analyzed using R software version 4.3.3 (12). Continuous variables were expressed as mean ± standard deviation or median (inter-quartile range) [*M* (*P*_25_, *P*_75_)] according to their distribution characteristics. Categorical variables were presented as frequencies and percentages [*n* (%)]. Comparisons of rates between groups, and between baseline and post-interventions were performed using Pearson's χ^2^ test and McNemar's test, respectively, with the significance level set at α = 0.05 (two-tailed).

## Results

### Baseline characteristics

The baseline characteristics of the study population are presented in Table 1. The cohort had a median age of 31 years, with a predominance of males (92.18%) and Han Chinese ethnicity (96.14%). The highest proportion of the population had completed high school or vocational school (52.36%), followed by college or higher education (38.40%). Marital status distribution showed that 53.14% were married and 44.58% single. About 48.33% never or occasionally smoked, 40.24% were current smokers, and 11.44% were former smokers. As shown, 79.21% never or occasionally drank, 17.08% were former drinkers, and only 3.70% were current drinkers. Regular exercise was reported by 72.92% of participants. Occupationally, firefighters constituted the largest group (66.6%), followed by bus drivers and port workers. Weekly working hours exceeded 56 h for 54.99% of participants, while 21.96% worked 40–48 h per week. The average working years was 4 years.

**Table 1:** Baseline characteristics of the study population (*n* = 3237).

**Variable**	**Value**
Age [year, *M* (*P*_25_, *P*_75_)]	31 (26, 38)
Gender (male, %)	2,984 (92.18)
Ethnicity (Han Chinese, %)	3,112 (96.14)
**Education [*****n*** **(%)]**	
Middle school or lower	299 (9.24)
High school or vocational school	1,695 (52.36)
College or higher	1,243 (38.40)
**Marital status [*****n*** **(%)]**	
Married	1,720 (53.14)
Single	1,443 (44.58)
Others^a^	74 (2.29)
**Smoking habit [*****n*** **(%)]**^b^	
Current smoker	1,214 (40.24)
Never or occasional smoker	1,458 (48.33)
Former smoker	345 (11.44)
**Drinking habit [*****n*** **(%)]**^c^	
Current drinker	96 (3.70)
Never or occasional drinker	2,054 (79.21)
Former drinker	443 (17.08)
**Regular exerciser [*****n*** **(%)]**^d^	
Yes	2,200 (72.92)
No	817 (27.08)
**Occupational category [*****n*** **(%)]**	
Firefighter	2,156 (66.60)
Bus driver	424 (13.10)
Video display terminal operator	220 (6.80)
Port worker	437 (13.50)
**Weekly working hours [*****n*** **(%)]**	
< 40 h	212 (6.55)
40–48 h	711 (21.96)
49–56 h	534 (16.50)
>56 h	1,780 (54.99)
Working years [year, *M* (*P*_25_, *P*_75_)]	4 (1, 10)

^a^Including divorced and widowed.

^b^Current smoker refers to an individual who smoked at least one cigarette per day for a minimum of 6 months prior to the survey, occasional smoker refers to an individual who reported smoking within the 6-month period preceding the survey but did not meet the criteria for current smoker, and former smoker refers to an individual with a history of smoking who abstained from smoking for at least 6 months prior to the survey.

^c^Current drinker refers to an individual who consumed alcohol at least once per week for a minimum of 6 months prior to the survey, occasional drinker refers to an individual who reported alcohol consumption within the 6-month period preceding the survey but did not meet the criteria for current drinker, and former drinker refers to an individual with a history of alcohol consumption who abstained from drinking for at least 6 months prior to the survey.

^d^Regular exerciser was defined as who engaged in at least moderate-intensity physical activity (resulting in noticeably accelerated breathing and heart rate) for ≥30 min per session, with a minimum frequency of twice weekly, sustained for 6 months or longer period prior to the survey.

### Baseline occupational stress detection rate, high social support rate, and comparative analysis by general characteristics

The baseline occupational stress detection rate in the cohort was 52.73%, while the high social support rate was 21.19%. Differences in occupational stress detection rates and high social support rates across various characteristics are presented in Table 2. As shown, the occupational stress detection rate was the lowest in the < 27 years group (49.24%) and the highest in the 27–34 years group (56.08%), with statistically significant difference between groups (*P* = 0.007). The high social support rate decreased with increasing age, showing statistically significant inter-group difference (*P* < 0.001). We found no statistically significant difference in either occupational stress detection rates or high social support rates between males and females. The Han population showed a higher occupational stress detection rate compared to other ethnicities (*P* = 0.046), while no significant differences were found in high social support rates across ethnicities. By education level, the highest occupational stress detection rate was observed in those with high school or vocational school education (57.52%), while the lowest was found in those with middle school education or lower (44.48%), with statistically significant inter-group difference (*P* < 0.001). The high social support rate increased with higher education levels, demonstrating statistically significant difference between groups (*P* = 0.045). No statistically significant differences in occupational stress detection rates were observed across marital status groups, but significant differences existed in high social support rates (*P* < 0.001). Current smokers exhibited the highest occupational stress detection rate (55.35%) and the lowest high social support rate (20.92%), whereas former smokers showed the lowest occupational stress detection rate (48.70%) and the highest high social support rate (23.48%). No statistically significant differences were found in either occupational stress detection rates or high social support rates among groups with different drinking habits. Although no significant difference in occupational stress detection rates was observed between regular exercisers and non-regular exercisers, the former group demonstrated significantly higher high social support rate (*P* = 0.018). While no statistically significant differences in occupational stress detection rates were found across occupational categories, firefighters showed significantly higher social support rate compared to other occupational groups (*P* = 0.023). The occupational stress detection rate increased with longer weekly working hours and longer working years (*P* < 0.001 and *P* = 0.046, respectively), whereas the high social support rate decreased with increasing weekly working hours and working years (*P* = 0.039 and *P* < 0.001, respectively).

**Table 2:** Baseline occupational stress detection rate, high social support rate, and comparative analysis by general characteristics.

**Variable**	**Sample size (*n)***	**Occupational stress**			**Social support**		
		**Occupational stress detection rate [*****n*** **(%)]**	χ^2^	***P***	**High social support rate [*****n*** **(%)]**	χ^2^	***P***
Total	3,237	1,707 (52.73)			686 (21.19)		
**Age (years)**			9.884	0.007		34.899	< 0.001
< 27	983	484 (49.24)			259 (26.35)		
27–34	1,118	627 (56.08)			246 (22.00)		
> 34	1,136	596 (52.46)			181 (15.93)		
**Gender**			0.536	0.464		1.046	0.306
Male	2,984	1,568 (52.55)			626 (20.98)		
Female	253	139 (54.94)			60 (23.72)		
**Ethnicity**			3.978	0.046		0.013	0.101
Han Chinese	3,112	1,652 (53.08)			659 (21.18)		
Others	125	55 (44.00)			27 (21.60)		
**Education**			22.476	< 0.001		6.206	0.045
Middle school or lower	299	133 (44.48)			54 (18.06)		
High school or vocational school	1,695	715 (57.52)			342 (20.18)		
College or higher	1,243	856 (50.68)			290 (23.33)		
**Marital status**			4.306	0.116		29.495	< 0.001
Married	1,720	915 (53.20)			310 (18.02)		
Single	1,443	745 (51.62)			367 (25.43)		
Others^a^	74	47 (63.51)			9 (12.16)		
**Smoking habit** ^b^			7.727	0.021		1.041	0.594
Current smoker	1,214	672 (55.35)			254 (20.92)		
Never or occasional smoker	1,458	740 (50.75)			314 (21.54)		
Former smoker	345	168 (48.70)			81 (23.48)		
**Drinking habit** ^c^			1.726	0.422		2.495	0.287
Current drinker	96	50 (52.08)			19 (19.79)		
Never or occasional drinker	2,054	1,086 (52.87)			444 (21.62)		
Former drinker	443	219 (49.44)			110 (24.83)		
**Regular exerciser** ^d^			1.753	0.186		5.607	0.018
Yes	2,200	1,136 (51.64)			497 (22.59)		
No	817	444 (54.35)			152 (18.60)		
**Occupational category**			4.428	0.219		9.538	0.023
Firefighter	2,156	1,140 (52.88)			490 (22.73)		
Bus driver	424	225 (53.07)			76 (17.92)		
Video display terminal operator	220	127 (57.73)			37 (16.82)		
Port worker	437	215 (49.20)			83 (18.99)		
**Weekly working hours**			38.883	< 0.001		8.348	0.039
< 40 h	212	79 (37.26)			51 (24.06)		
40–48 h	711	341 (47.96)			173 (24.33)		
49–56 h	534	279 (51.69)			115 (21.54)		
>56 h	1,780	1,011 (56.80)			347 (19.49)		
**Working years**			6.146	0.046		27.371	< 0.001
< 2 y	819	416 (50.79)			218 (26.62)		
2–6 y	1,253	643 (51.32)			271 (21.63)		
>6 y	1,165	648 (55.62)			197 (16.91)		

^a^Including divorced and widowed.

^b^Current smoker refers to an individual who smoked at least one cigarette per day for a minimum of 6 months prior to the survey, occasional smoker refers to an individual who reported smoking within the 6-month period preceding the survey but did not meet the criteria for current smoker, and former smoker refers to an individual with a history of smoking who abstained from smoking for at least 6 months prior to the survey.

^c^Current drinker refers to an individual who consumed alcohol at least once per week for a minimum of 6 months prior to the survey, occasional drinker refers to an individual who reported alcohol consumption within the 6-month period preceding the survey but did not meet the criteria for current drinker, and former drinker refers to an individual with a history of alcohol consumption who abstained from drinking for at least 6 months prior to the survey.

^d^Regular exerciser was defined as who engaged in at least moderate-intensity physical activity (resulting in noticeably accelerated breathing and heart rate) for ≥30 min per session, with a minimum frequency of twice weekly, sustained for 6 months or longer period prior to the survey.

### Changes in occupational stress detection rate and high social support rate following mental health interventions

**Figure 1:**
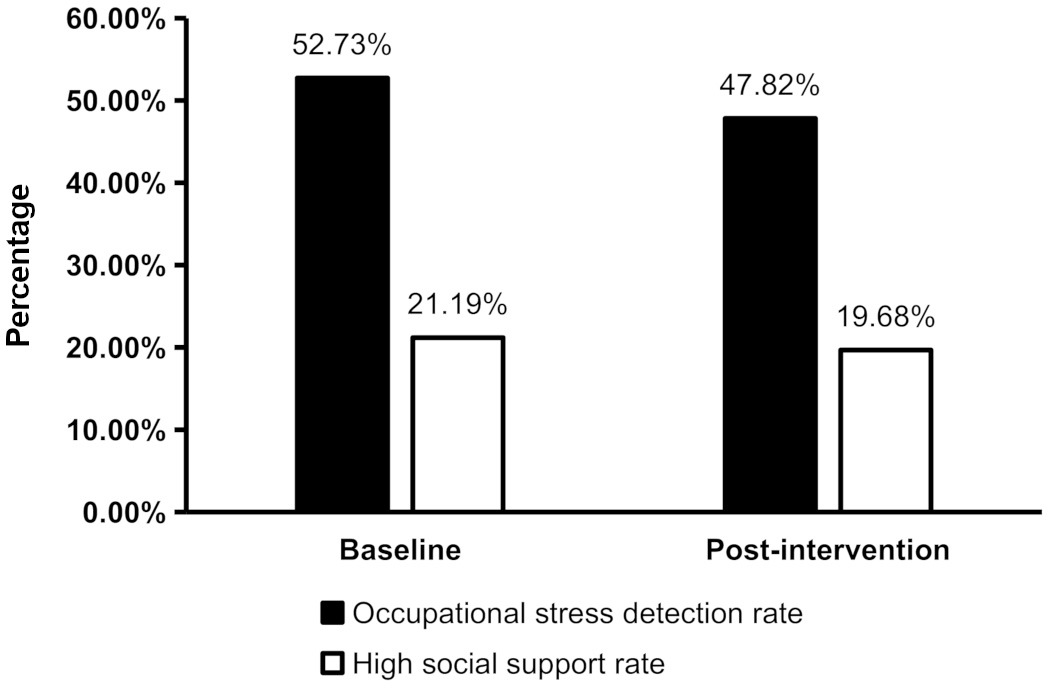
Changes in occupational stress detection rate and high social support rate following mental health interventions (*n* = 3237). The occupational stress detection rate after the interventions showed a statistically significant reduction compared to the baseline rate (χ^2^ = 21.443, *P* < 0.001), while the high social support rate was not statistically different from the baseline rate (χ^2^ = 3.258, *P* = 0.071).

After the 2 years of mental health interventions, the occupational stress detection rate in the cohort was 47.82%, demonstrating a statistically significant reduction compared to the baseline rate (χ^2^ = 21.443, *P* < 0.001). In contrast, the high social support rate measured 19.68%, showing no statistically significant difference from the baseline rate (χ^2^ = 3.258, *P* = 0.071) (Figure 1).

### Comparison of baseline and post-intervention occupational stress detection rates stratified by general characteristics

The comparative results of occupational stress detection rates before and after the psychological interventions, categorized by general characteristics of the cohort, are presented in Table 3. Statistically significant reductions in occupational stress detection rates after the interventions (all *P* < 0.05) were observed among individuals aged ≥ 27 years, male participants, Han Chinese, those with high school or vocational school education or higher, married individuals, current smokers, never or occasional smokers, never or occasional drinkers, firefighters and bus drivers, workers with weekly working hours of 40–48 h or >56 h, and those with >6 working years. Conversely, no statistically significant differences in occupational stress detection rates were found between baseline and post-interventions for individuals aged < 27 years, female participants, non-Han ethnic groups, those with middle school education or lower, single or other marital statuses, former smokers, current or former drinkers, VDT operators and port workers, and those with weekly working hours < 40 h or 49–56 h and working years ≤ 6 years.

**Table 3:** Comparison of baseline and post-intervention occupational stress detection rates stratified by general characteristics.

**Variable**	***n***	**Baseline rate (%)**	**Post-intervention rate (%)**	**χ^2^**	***P***
**Age (years)**					
< 27	983	49.24	48.22	0.266	0.606
27–34	1,118	56.08	51.16	7.619	0.006
>34	1,136	52.46	44.19	21.764	< 0.001
**Gender**					
Male	2,984	52.55	47.22	22.921	< 0.001
Female	253	54.94	54.94	0.000	1.000
**Ethnicity**					
Han Chinese	3,112	53.08	47.94	22.496	< 0.001
Others	125	44.00	44.80	0.024	0.876
**Education**					
Middle school or lower	299	44.48	41.47	0.742	0.389
High school or vocational school	1,695	50.68	44.31	18.056	< 0.001
College or higher	1,243	57.52	54.14	4.161	0.041
**Marital status**					
Married	1,720	53.20	46.80	19.967	< 0.001
Single	1,443	51.63	48.93	2.811	0.094
Others^a^	74	63.51	50.00	3.125	0.077
**Smoking habit** ^b^					
Current smoker	1,214	55.35	47.61	19.991	< 0.001
Never or occasional smoker	1,458	50.75	47.6	3.861	0.049
Former smoker	345	48.70	44.06	2.098	0.148
**Drinking habit** ^c^					
Current drinker	96	52.08	46.88	1.000	0.317
Never or occasional drinker	2,054	52.87	49.85	5.139	0.023
Former drinker	443	49.44	44.02	3.349	0.067
**Regular exerciser** ^d^					
Yes	2,200	51.64	47.55	9.831	0.002
No	817	54.35	46.27	15.125	< 0.001
**Occupational category**					
Firefighter	2,156	52.88	48.61	10.424	0.001
Bus driver	424	53.07	37.74	25.299	< 0.001
Video display terminal operator	220	57.73	56.36	0.134	0.714
Port worker	437	49.20	49.43	0.008	0.931
**Weekly working hours**					
< 40 h	212	37.26	36.32	0.054	0.816
40–48 h	711	47.96	43.46	3.879	0.049
49–56 h	534	51.69	48.69	1.347	0.246
>56 h	1,780	56.80	52.67	18.251	< 0.001
< 2 y	819	50.79	48.23	1.485	0.223
2–6 y	1,253	51.32	48.28	3.126	0.077
>6 y	1,165	55.62	47.04	23.809	< 0.001

^a^Including divorced and widowed.

^b^Current smoker refers to an individual who smoked at least one cigarette per day for a minimum of 6 months prior to the survey, occasional smoker refers to an individual who reported smoking within the 6-month period preceding the survey but did not meet the criteria for current smoker, and former smoker refers to an individual with a history of smoking who abstained from smoking for at least 6 months prior to the survey.

^c^Current drinker refers to an individual who consumed alcohol at least once per week for a minimum of 6 months prior to the survey, occasional drinker refers to an individual who reported alcohol consumption within the 6-month period preceding the survey but did not meet the criteria for current drinker, and former drinker refers to an individual with a history of alcohol consumption who abstained from drinking for at least 6 months prior to the survey.

^d^Regular exerciser was defined as who engaged in at least moderate-intensity physical activity (resulting in noticeably accelerated breathing and heart rate) for ≥30 min per session, with a minimum frequency of twice weekly, sustained for 6 months or longer period prior to the survey.

## Discussion

The study investigated occupational stress among 3,237 high-risk workers including firefighters, bus drivers, VDT operators and port workers in Shenzhen, China, and evaluated the effectiveness of mental health interventions subsequently implemented in a period of about 2 years. The results revealed that the baseline occupational stress detection rate among the cohort population reached 52.73%, indicating that more than half of the high-risk workers experienced occupational stress, suggesting a substantial occupational stress burden in Shenzhen, China. Compared with previous relevant studies (13–15), the detection rate was generally at a higher level, which may be associated with certain demographic characteristics, lifestyle habits, and occupational factors within this population.

Further detailed analysis revealed significant variations in occupational stress detection rates across different age groups, ethnicities, educational levels, smoking habits, weekly working hours, and working years. Specifically, the highest occupational stress detection rate was observed in the 27–34 age group, consistent with the previous report (16). This may be attributed to individuals of this age group being at a critical career development stage, facing multiple challenges including job pressure and career advancement (16). The Han Chinese ethnic group demonstrated higher occupational stress detection rates compared to other ethnicities, aligning with findings from Lian et al. (17, 18). This phenomenon may be associated with potential differences in occupational distributions and job demands among various ethnicities. Individuals with higher education levels exhibited relatively elevated occupational stress detection rates, potentially due to greater career expectations and intensified workplace competition. However, the relationship between education level and occupational stress remains inconsistent in literature (19–21). Current smokers showed higher occupational stress detection rates, possibly because smoking serves as a convenient yet unhealthy coping mechanism for work-related stress, corroborating previous relevant studies (22–24). We found that minimal differences were observed across occupational categories, suggesting that all professions in this region might be similarly influenced by local economic conditions and societal rhythms. Moreover, occupational stress detection rates increased with both weekly working hours and working years, confirming prolonged work as a significant risk factor for occupational stress, as previously documented (25, 26).

The baseline high social support rate in the cohort was only 21.19%, indicating a relatively low level. Social support plays a crucial role in mitigating occupational stress, and low social support may hinder workers' ability to cope with work-related pressure (27, 28). Significant variations in high social support rates were observed across different age groups, educational levels, marital statuses, exercise habits, occupational categories, weekly working hours, and working years. Specifically, younger participants (< 27 years) exhibited higher high social support rates, potentially attributable to their recent entry into the workforce, more active social networks, and stronger family support systems (29). Higher education levels correlated with increased social support, possibly reflecting greater social recognition and access to support resources (30). Married individuals showed lower high social support rates than their unmarried counterparts, likely due to the competing demands of work-family balance reducing available support (31). Regular exercisers demonstrated higher high social support, suggesting that physical activity may enhance social connectivity in addition to its physiological benefits (32). Firefighters displayed notably higher high social support, potentially associated with the collective organizational culture inherent in this profession. A progressive decline in high social support was observed with increasing weekly working hours and longer working years, possibly resulting from accumulated workplace conflicts, communication breakdowns, and competitive pressures over time.

The study found a significant reduction in occupational stress detection rates among the cohort following mental health interventions, suggesting that regular occupational mental health training and group psychological support sessions could be effective to some extent in alleviating occupational stress. The intervention effects varied across subgroups stratified by general characteristics, with more pronounced reductions observed in participants aged ≥ 27 years, male individuals, Han ethnicity, those with high school or vocational school education or higher, married participants, current smokers, never or occasional smokers, never or occasional drinkers, firefighters and bus drivers, workers with weekly working hours of 40–48 h or >56 h, and those with working years > 6 years. This differential effectiveness may be attributed to the fact that these subgroups face more pronounced occupational stressors in their professional lives, making the psychological coping skills and support gained through the interventions particularly beneficial for the stress reduction.

However, the change in high social support rate post-intervention did not reach statistical significance. This may be attributable to insufficient focus on social support enhancement in the current intervention measures, such as mental health training and group psychological support sessions. Future interventions could incorporate strategies to expand social networks and establish workplace support groups, which may further improve workers' social support levels.

The study has several limitations that should be acknowledged. First, the study implemented stringent inclusion criteria for participants to meet intervention requirements, with the study population primarily drawn from four high-risk occupational groups: firefighters, bus drivers, VDT operators, and port workers. Notably, firefighters constituted over 50% of the cohort. This sampling approach may introduce selection bias, consequently limiting the generalizability of the research findings. Second, the intervention approach was predominantly limited to mental health training and group psychological support sessions. While these demonstrated significant effectiveness, the singular focus may have constrained the comprehensiveness of intervention outcomes. What is more, the reliance on questionnaire-based data collection introduces the possibility of information bias, which may affect the accuracy of the reported results.

Future research could expand the scope of study participants, adopt comprehensive intervention models combining multiple approaches, optimize strategies for different occupational groups, and incorporate objective data such as physiological indicators for more holistic evaluation. Meanwhile, further in-depth investigation into the mechanisms through which various population characteristics influence occupational stress and social support should be conducted to provide a theoretical basis for developing more precise and effective occupational health promotion strategies.

## Data Availability

The original contributions presented in the study are included in the article/Supplementary material, further inquiries can be directed to the corresponding authors.
